# Growth promotion and protective potentials of leaf infusions of *Parkia biglobosa*, *Moringa oleifera* and *Vernonia amygdalina* on *Abelmoschus esculentus* seeds

**DOI:** 10.1038/s41598-022-18555-4

**Published:** 2022-08-18

**Authors:** Oghenerobor B. Akpor, Marvellous-Mercy Maxwell, Ikponmwosa O. Evbuomwan, Tomilola D. Olaolu, Abigail G. Adeyonu, Omorefosa O. Osemwegie

**Affiliations:** 1 Department of Biological Sciences, College of Sciences, Afe Babalola University, PMB 5454, Ado-Ekiti, Ekiti State Nigeria; 2 Department of Food Science and Microbiology, College of Pure and Applied Sciences, Landmark University, PMB 1001, Omu-Aran, Kwara State Nigeria; 3 Department of Biochemistry, College of Pure and Applied Sciences, Landmark University, PMB 1001, Omu-Aran, Kwara State Nigeria; 4 Department of Agriculture, College of Agricultural Sciences, Landmark University, PMB 1001, Omu-Aran, Kwara State Nigeria

**Keywords:** Biotechnology, Microbiology, Plant sciences

## Abstract

The germinability and protective potential of leaf infusion of *Parkia biglobosa, Moringa oleifera* and *Vernonia amygdalina* leaves on okra (*Abelmoschus esculentus*) seeds against infections simulated with suspended cells of *Aspergillus niger, A. flavus, A. fumigatus,* and *Penicillium* sp. were examined. Prior to planting, the okra seeds were first surface-sterilized in 5% sodium hypochlorite solution before steeping in known concentrations (0, 20, 40, 60, 80, and 100%) of the respective leaf infusions for a known duration. Seven of the steeped seeds were planted in plastic transparent containers, incubated for 7 days under light, and observed daily. Germination index, germination rate, germination time, and vigor index were calculated for each treatment, using standard procedures. The effective concentrations of the infusions of *V. amygdalina*, *P. biglobosa* and *M. oleifera* were 40, 40, and 60% respectively. Optimum steeping durations in leaf infusions were 1, 5, and 6 h, for *P. biglobosa*, *M. oleifera* and *V. amygdalina*, respectively. All the leaf infusions were observed to protect the okra seeds against infections with the test organisms. Furthermore, seeds steeped in the respective leaf infusions showed remarkably higher germinability potential than the control seeds steeped in water. The study confirmed that the leaf infusions may be attractive as economic alternatives for seed priming and protection.

## Introduction

The treatment of seeds is known to improve their germination performance and enhance vigor. One method that has been established to improve the seedling quality and vigor is seed priming, which is reported to improve respiratory activities of seedlings. Other benefits of seed priming include improvement of seed emergence speed, enhancement of uniformity, hence improving harvesting productivity, increase in yield potential and improvement of vigor^1–3^.

Seed priming have several benefits, such as improvement of seed emergence speed, enhancement of uniformity, hence improving harvesting productivity, increase in yield potential and improvement of vigor by making the plant stronger. Although several seed priming techniques (chemical, hydro, osmo, bio, nutrient-priming, use of plant growth regulators, plant extracts, nanoparticles and physical agents) are reported in literatures, none is without limitations^2,4,5^. In addition, not all priming methods may lead to significant germination and growth. This is because the use of inappropriate priming conditions or technique may lead to loss of seed tolerance to desiccation, thus reducing viability reduces seed viability. It is therefore advocated that identifying the ideal priming method for a plant is vital in enhancing germination and growth^2,6^. Besides, the use of a seed primer that covers additional protection against plant pathogens is an added advantage. It has been indicated that plant extracts can play vital role in protecting the plants against microbial pathogens^3,7^.

The common method for dealing with a large number of plant diseases is ab initio the preventive method. An integration of plant disease management strategies which involves cultural practices, sanitation and seasonal applications of spray have now included the pre-treatment of seeds before planting and the use of resistant variety^8,9^. Proper handling and treatment of seeds help in the improvement of seed quality and also contribute significantly to increase crop yield. The use of chemicals such as pesticides is a primary pest control measure used by farmers^10^. Because most bioactive chemicals of plant origin are preponderant and circulate effectively along the ecological food chain, they have become a readily available bioresource for trial in the search for non-chemical, eco-friendly, less expensive, and non-toxic alternative in seeds treatment processes.

Globally, the prevention of plant pests and diseases has been a major challenge threatening humans’ food security^11^. Although agrochemicals are hitherto used for seed treatments, diseases and pest prevention, they were reported to pose several chronic threats to animals, humans and the environment either directly or indirectly^12^. According to Sharma et al.^13^, agrochemicals have been linked to several illnesses, such as cancer, asthma, hormone disruption particularly among farmers^14^. The persistent use of these chemicals also leads to the depletion of beneficial microorganisms in the plant soil environment. Therefore, the use of eco-friendly biochemicals that can suppress pathogens and effect healthy germination of seeds are advocated. Medicinal plants indicated to have antimicrobial and seed priming potentials, hence could serve as good alternative to agrochemicals^15^. This study was therefore aimed at investigating the effect of the leaf infusions of *Parkia biglobosa, Moringa oleifera* and *Vernonia amygdalina* on germinability and protection of okra (*Abelmoschus esculentus*) seeds against selected fungal pathogens.

## Results

### Phytochemical composition of the leaf infusions

In all the leaf infusion samples, terpenoids and alkaloids were detected while phlobatannins, anthraquinones and steroids were absent (Table 1).

**Table 1:** Phytochemical compositions of leaf infusions.

Phytochemical	*Parkia biglobosa*	*Moringa oleifera*	*Vernonia amygdalina*
Saponins	–	+	+
Cardiac glycosides	–	+	+
Anthraquinones	–	–	–
Terpenoids	+	+	+
Alkaloids	+	+	+
Phlobatannins	–	+	–
Flavonoids	–	–	–
Steroids	–	–	–

(+) and (–) represent presence and absence of phytochemical compositions in the extracts.

### Effect of leaf infusion concentration on germinability

When the seeds were steeped in the *V. amygdalina* before planting, higher percentage germination of 71.43% was observed for the 40, 60 and 80% dilutions respectively. Vigor index was also observed to be higher for seeds steeped in the 40% dilution of the same plant. Generally, germination rate and time of seeds steeped in the different dilutions and control setups ranged between 0.18 and 0.19; and 5.16 and 5.38 respectively (Table 2).

**Table 2:** Germinability of the okra seeds when steeped at different concentrations of the respective leaf infusions before planting.

	% Germination	Germination index	Germination rate	Germination time	Vigor index
***Parkia bigblobosa***					
100%	64.29 (± 10.10)	59.65 (± 15.74)	5.61 (± 0.68)	0.18 (± 0.02)	559.2 (± 305.93)
80%	42.86 (± 0.00)	38.50 (± 0.00)	5.23 (± 0.00)	0.19 (± 0.00)	505.10 (± 194.9)
60%	21.43 (± 10.10)	27.44 (± 5.35)	5.25 (± 0.35)	0.19 (± 0.01)	31.63 (± 18.76)
40%	64.29 (± 10.10)	70.88 (± 0.54)	5.07 (± 0.09)	0.20 (± 0.00)	606.12 (± 251.10)
20%	71.43 (± 0.00)	72.41 (± 1.26)	5.11 (± 0.03)	0.20 (± 0.00)	836.80 (± 86.58)
Control	28.57 (± 0.00)	22.18 (± 1.01)	5.40 (± 0.04)	0.19 (± 0.00)	106.12 (± 23.1)
***Moringa oleifera***					
100%	42.86 (± 0.00)	45.65 (± 1.68)	5.07 (± 0.10)	0.20 (± 0.00)	688.8 (± 99.57)
80%	35.71 (± 30.30)	36.10 (± 1.91)	5.22 (± 0.60)	0.19 (± 0.02)	132.7 (± 25.98)
60%	57.14 (± 20.20)	49.88 (± 11.04)	5.11 (± 0.04)	0.20 (± 0.00)	837.8 (± 734.53)
40%	28.57 (± 0.00)	27.95 (± 4.63)	5.36 (± 0.51)	0.19 (± 0.02)	169.4 (± 60.61)
20%	42.86 (± 20.20)	53.45 (± 12.72)	5.11 (± 0.16)	0.20 (± 0.01)	477.6 (± 525.3)
Control	28.57 (± 0.00)	22.18 (± 1.01)	5.40 (± 0.04)	0.19 (± 0.00)	106.12 (± 23.1)
***Vernonia amygdalina***					
100%	50.00 (± 10.10)	43.96 (± 2.57)	5.30 (± 0.10)	0.19 (± 0.00)	439.80 (± 93.80)
80%	57.14 (± 20.20)	61.33 (± 16.93)	5.44 (± 0.51)	0.18 (± 0.02)	425.51 (± 350.7)
60%	35.71 (30.30)	43.30 (± 15.30)	5.33 (± 0.24)	0.19 (± 0.01)	417.4 (± 529.61)
40%	64.29 (± 10.10)	64.27 (± 2.57)	5.07 (± 0.09)	0.20 (± 0.00)	680.61 (± 235.22)
20%	50.00 (± 30.30)	44.78 (± 13.44)	5.13 (± 0.00)	0.20 (± 0.00)	786.73 (± 864.40)
Control	28.57 (± 0.00)	22.18 (± 1.01)	5.40 (± 0.04)	0.19 (± 0.00)	106.12 (± 23.1)

Values are averages of duplicates with those in parenthesis representing ± standard deviation. % values indicate the dilution of the leaf infusion in water while control represents seeds steeped in distilled water.

In the presence of the respective concentrations of the *P. biglobosa* leaf infusions, the germination rate and time of the okra seeds showed no observable difference, ranging from 0.19 to 0.20 day^−1^ and from 5.40 to 5.61 day^−7^ respectively, across the treatments. The best percentage germination results of 71.43% and vigor index of 836.80 was however observed in seeds that were steeped in 20% dilution of the *P. biglobosa* (Table 2). For seeds that were steeped in the *M. oleifera* leaf infusion, highest percentage germination (57.14%) and vigor index (837) were observed in seeds treated with 60% of the infusion. Across the different treatments, germination rate and time ranged from 0.18–0.20 day^−1^ to 5.22–5.36 day^−1^ respectively were recorded (Table 2). In the case of seeds that were treated with the *V. amygdalina* leaf infusion, highest vigor index of 786.73 was observed in the 20% infusion treated seeds while the highest percentage germination of 57.14% was observed for the 80% infusion treated seeds. Germination time that ranged from 5.40 to 5.44 day was observed across the respective treatments while germination rate across the respective treatments ranged between 0.18 and 0.20 day^−1^ (Table 2).

When the effect of different levels of concentration of the respective leaf infusions was considered, it was observed that 20% level of concentration gave the highest germination index (56.88) and this is statistically significantly different from 0, 60 and 100% concentration levels respectively. Also, 100, 80, 60 and 40% concentration levels significantly compared with 0% level of concentration. When considered as individual factors, leaf infusion and concentration level significantly influenced germination index of okra at 100%. Likewise, the relationship between interaction of the two were also significant on the germination index of okra at 100%. However, the respective infusions had no significant influence on the vigour index of okra. While 20% level of concentration gave the highest mean value of vigour index, the control setup (seeds steeped in water before planting) had the least and also significantly differed from others (Table 3).

**Table 3:** Effects of level of concentration of the leaf infusions on germination and vigor indices of okra.

Leaf infusion type (LT)	Germination index	Vigour index
Locust beans	48.51a	440.82a
Moringa	39.00b	402.04a
Bitter leaf	46.69a	479.76a
**Concentration level (CL)**		
100%	49.75bc	562.58ab
80%	45.31ab	354.41b
60%	40.20d	428.91b
40%	54.36ab	485.37ab
20%	56.88a	700.34a
0%	21.89e	113.61c
LT	0.000	Ns
CL	0.000	0.000
LT*CL	0.000	0.004

Note: Values followed by similar letters under the same column are not significantly different at p = 0.05 according to Duncan’s multiple range test; ns = not significant at p = 0.05 level.

### Effect of steeping duration in the leaf infusions on germinability

Seeds that were steeped for 6 h in the *P. biglobosa* leaf infusion before planting were observed to show significantly higher germinability and vigor index than seeds that were steeped at other durations. Germination rate and time that ranged from 0.17 to 0.20 day^−1^ and from 4.97 to 6.32 day were observed for seeds across the different soaking durations (Table 4).

**Table 4:** Germinability of the okra seeds at the respective steeping durations in the leaf infusions before planting.

Steeping duration	% Germination	Germination index	Germination time	Germination rate	Vigor index
***Parkia bigblobosa***					
1 h	42.86 (± 20.20)	31.67 (± 11.88)	5.99 (± 0.96)	0.17 (± 0.03)	404.08 (± 525.28)
2 h	21.43 (± 10.10)	11.96 (± 1.56)	5.67 (± 0.24)	0.18 (± 0.01)	43.88 (± 38.96)
3 h	28.57 (± 0.00)	35.39 (± 4.21)	4.97 (± 0.35)	0.20 (± 0.01)	208.16 (± 202.03)
4 h	14.29 (± 0.00)	11.15 (± 5.47)	5.25 (± 1.06)	0.19 (± 0.04)	7.14 (± 4.33)
5 h	28.57 (± 20.20)	8.78 (± 9.52)	6.32 (± 0.96)	0.16 (± 0.02)	79.59 (± 86.58)
6 h	50.00 (± 30.30)	66.11 (± 27.00)	5.69 (± 1.14)	0.18 (± 0.04)	874.49 (± 1000.05)
***Moringa oleifera***					
1 h	35.71 (± 10.10)	59.59 (± 0.72)	4.57 (± 0.10)	0.22 (± 0.00)	360.20 (± 379.53)
2 h	42.86 (± 0.00)	39.40 (± 8.00)	5.08 (± 0.47)	0.20 (± 0.02)	385.71 (± 233.78)
3 h	21.43 (± 10.10)	11.72 (± 10.32)	5.70 (± 1.13)	0.18 (± 0.04)	39.80 (± 53.39)
4 h	14.29 (± 0.00)	3.83 (± 0.84)	6.75 (± 0.35)	0.15 (± 0.01)	12.24 (± 11.54)
5 h	50.00 (± 10.10)	64.68 (± 10.32)	4.90 (± 0.13)	0.20 (± 0.01)	769.39 (± 655.16)
6 h	35.71 (± 10.10)	50.08 (± 5.52)	4.77 (± 0.06)	0.21 (± 0.00)	154.08 (± 50.51)
***Vernonia amygdalina***					
1 h	21.43 (± 10.10)	29.29 (± 12.84)	5.86 (± 1.61)	0.18 (± 0.05)	418.37 (± 562.80)
2 h	28.57 (± 20.20)	30.64 (± 7.08)	5.12 (± 0.18)	0.20 (± 0.01)	281.63 (± 294.39)
3 h	28.57 (± 0.00)	43.72 (± 2.53)	4.61 (± 0.16)	0.22 (± 0.01)	224.49 (± 0.00)
4 h	42.86 (± 20.20)	39.80 (± 12.13)	4.92 (± 0.11)	0.20 (± 0.00)	240.82 (± 144.31)
5 h	42.86 (± 20.20)	34.27 (± 11.04)	5.14 (± 0.12)	0.19 (± 0.00)	497.96 (± 531.05)
6 h	50.00 (± 10.10)	77.89 (± 18.57)	4.87 (± 0.52)	0.21 (± 0.02)	345.92 (± 203.47)
**Control**					
1 h	42.86 (± 40.41)	28.55 (± 18.29)	5.09 (± 0.13)	0.20 (± 0.00)	666.33 (± 904.81)
2 h	35.71 (± 30.30)	38.24 (10.67)	5.19 (± 0.27)	0.19 (± 0.01)	348.98 (476.21)
3 h	57.14 (± 20.20)	55.83 (± 9.36)	4.99 (± 0.08)	0.20 (± 0.00)	739.80 (± 483.43)
4 h	50.00 (± 30.30)	47.10 (± 19.08)	4.87 (± 0.04)	0.21 (± 0.00)	602.04.7 (± 476.2)
5 h	50.00 (± 10.10)	59.86 (± 8.05)	4.92 (± 0.11)	0.20 (± 0.00)	657.14.7 (± 132.8)
6 h	64.29 (± 30.30)	91.49 (± 19.93)	4.89 (± 0.07)	0.20 (± 0.00)	1129.6 (± 809.6)

In the presence of the *M. oleifera* leaf infusion, 5 h steeping time was observed to show highest germinability at the end of the planting period. A highest vigor index of 769.40 was also observed for seeds that were steeped for 5 h before planting. Germination and vigor index values for seeds that were steeped for 5 h before planting were shown to be significantly higher than values recorded for other steeping durations (Table 4). For seeds that were steeped in the *V. amygdalina* leaf infusion before planting, highest germination and vigor index were recorded in seeds that were steeped at 6 h and 5 h, respectively. Generally, germination observed for seeds steeped for 6 h before planting was significantly higher than those observed for seeds steeped at other durations (Table 4). The seeds soaked in water only revealed 6 h to be the highest having the vigor and germination index of 1129.6 and 91.49 respectively (Table 4).

The *M. oleifera* leaf infusion had the highest germination index (55.58) which was statistically significant from those of *P. biglobosa* (27.51) and *V. amygdalina* (42.60) but not significantly different from control (water). Considering the effect of soaking time, a highest germination index (97.39) mean value was observed at 6 h, which was statistically significantly different from all other treatments. The 4 h steeping duration treatment with mean value of 25.47 had the least germination index which was significantly different from others. The mean germination index at 5 h, 1 h and 3 h were 41.89, 37.27and 36.67, respectively and were not observed to be statistically different from one another. Results show that when examined as individual factors, type of leave infusion and steeping duration infusion time significantly determined germination index of okra at 1 and 5% levels respectively.

The interactive effect of type of leaf infusion and steeping duration was also significant at 1% level of significance. Furthermore, the analysis showed that water had the highest vigor index (690.64) and was statistically significantly different from others. Regarding the soaking time, 6 h had a vigour index mean value of 600.02 and significantly compared with 4, 3 and 2 h treatments respectively (Table 5).

**Table 5:** Effect of leave infusion of *P. biglobosa*, *M. oleifera* and *V. amygdalina*on germination and vigor indices of okra.

Type leave infusion (TLI)	Germination index	Vigour index
Locust beans	27.51c	269.56b
Moringa	55.58a	269.57b
Bitter leaf	42.60b	334.87b
Water	53.51a	690.64a
**Steeping duration (SD)**		
1 h	37.27bc	462.25ab
2 h	30.06cd	265.05b
3 h	36.67bc	303.66b
4 h	25.47d	215.56b
5 h	41.89b	501.02ab
6 h	97.39a	600.02a
TLI	0.000	0.000
SD	0.012	0.026
TLI*SD	0.000	Ns

Note: Values followed by similar letters under the same column are not significantly different at p = 0.05 according to Duncan’s multiple range test; ns = not significant at p = 0.05 level.

**Table 6:** Protective potential of leaf infusion from the *P. biglobosa*.

Treatments	Germination	Index	Time	Rate	Vigor
**Infected and untreated**					
*Aspergillus flavus*	7.14 (± 10.10)	36.05 (± 3.1)	3.86 (± 0.20)	0.26 (± 0.01)	19.39 (± 27.42)
*Aspergillus niger*	7.14 (± 10.10)	39.77 (± 4.8)	3.79 (± 0.49)	0.27 (± 0.03)	13.27 (± 18.76)
*Aspergillus fumigatus*	0.00 (± 0.00)	32.68 (± 2.3)	3.77 (± 0.15)	0.27 (± 0.01)	0.00 (± 0.00)
*Penicillium* sp.	7.14 (± 0.00)	18.67 (± 5.77)	4.50 (± 0.00)	0.22 (± 0.00)	6.12 (± 8.66)
**Infected, then treated**					
*Aspergillus flavus*	64.29 (± 10.10)	31.47 (± 9.73)	5.65 (± 0.50)	0.18 (± 0.02)	505.1 (± 198.4)
*Aspergillus niger*	57.14 (± 20.20)	32.98 (± 15.42)	5.34 (± 0.41)	0.19 (± 0.01)	486.7 (± 350.67)
*Aspergillus fumigatus*	50.00 (± 30.30)	71.35 (± 12.18)	4.68 (± 0.26)	0.21 (± 0.01)	433.7 (± 497.9)
*Penicillium* sp.	57.14 (± 40.41)	71.99 (± 12.48)	4.79 (± 0.41)	0.21 (± 0.02)	634.7 (± 487.8)
**Treated, then infected**					
*Aspergillus niger*	0.00 (± 0.00)	14.58 (± 3.8)	2.62 (± 0.18)	0.38 (± 0.03)	0.00 (± 0.00)
*Aspergillus flavus*	0.00 (± 0.00)	19.64 (± 9.26)	0.00 (± 0.00)	0.00 (± 0.00)	0.00 (± 0.00)
*Aspergillus fumigatus*	7.14 (± 10.10)	33.30 (± 7.03)	3.75 (± 0.35)	0.27 (± 0.03)	2.04 (± 2.89)
*Penicillium* sp.	0.00 (± 0.00)	21.73 (± 1.26)	2.54 (± 0.29)	0.40 (± 0.05)	0.00 (± 0.00)
**Infected, then steeped in water**					
*Aspergillus niger*	14.29 (± 20.20)	41.56 (± 1.50)	4.16 (± 0.80)	0.24 (± 0.05)	38.78 (± 54.84)
*Aspergillus flavus*	21.43 (± 10.10)	46.36 (± 6.01)	4.49 (± 0.58)	0.22 (± 0.03)	57.14 (± 51.95)
*Aspergillus fumigatus*	28.57 (± 0.00)	38.66 (± 11.4)	5.27 (± 1.03)	0.19 (± 0.04)	53.06 (± 28.86)
*Penicillium* sp.	14.29 (± 20.20)	41.50 (± 16.93)	4.00 (± 0.40)	0.25 (± 0.03)	26.53 (± 37.52)

Germination, index, time, rate and vigor represent % germination, germination index, germination time, germination rate and vigor, respectively.

**Table 7:** Protective potential of leaf infusion from the *M. oleifera*.

Treatments	Germination	Index	Time	Rate	Vigor
**Infected and untreated**					
*Aspergillus flavus*	7.14 (± 10.10)	36.05 (± 3.1)	3.86 (± 0.20)	0.26 (± 0.01)	19.39 (± 27.42)
*Aspergillus niger*	7.14 (± 10.10)	39.77 (± 4.8)	3.79 (± 0.49)	0.27 (± 0.03)	13.27 (± 18.76)
*Aspergillus fumigatus*	0.00 (± 0.00)	32.68 (± 2.3)	3.77 (± 0.15)	0.27 (± 0.01)	0.00 (± 0.00)
*Penicillium* sp.	7.14 (± 0.00)	18.67 (± 5.77)	4.50 (± 0.00)	0.22 (± 0.00)	6.12 (± 8.66)
**Infected, then treated**					
*Aspergillus flavus*	64.29 (± 10.10)	51.17 (± 6.85)	5.36 (± 0.60)	0.19 (± 0.02)	536.7 (± 251.1)
*Aspergillus niger*	57.14 (± 0.00)	67.15 (± 5.64)	4.78 (± 0.00)	0.21 (± 0.00)	273.5 (± 109.7)
*Aspergillus fumigatus*	57.14 (± 0.00)	85.13 (± 7.32)	4.77 (± 0.12)	0.21 (± 0.01)	1036.3 (± 323.8)
*Penicillium* sp.	385.7 (± 444.4)	243.7 (± 255.8)	5.01 (± 0.06)	0.20 (± 0.00)	4928.5 (± 6464.9)
**Treated, then infected**					
*Aspergillus niger*	0.00 (± 0.00)	5.36 (± 2.53)	0.00 (± 0.00)	0.00 (± 0.00)	0.00 (± 0.00)
*Aspergillus flavus*	0.00 (± 0.00)	17.86 (± 8.42)	0.00 (± 0.00)	0.00 (± 0.00)	0.00 (± 0.00)
*Aspergillus fumigatus*	0.00 (± 0.00)	6.37 (± 9.01)	0.00 (± 0.00)	0.00 (± 0.00)	0.00 (± 0.00)
*Penicillium* sp.	0.00 (± 0.00)	8.93 (4.21)	0.00 (± 0.00)	0.00 (± 0.00)	0.00 (± 0.00)
**Infected, then steeped in water**					
*Aspergillus niger*	14.29 (± 20.20)	41.56 (± 1.50)	4.16 (± 0.80)	0.24 (± 0.05)	38.78 (± 54.84)
*Aspergillus flavus*	21.43 (± 10.10)	46.36 (± 6.01)	4.49 (± 0.58)	0.22 (± 0.03)	57.14 (± 51.95)
*Aspergillus fumigatus*	28.57 (± 0.00)	38.66 (± 11.4)	5.27 (± 1.03)	0.19 (± 0.04)	53.06 (± 28.86)
*Penicillium* sp.	14.29 (± 20.20)	41.50 (± 16.93)	4.00 (± 0.40)	0.25 (± 0.03)	26.53 (± 37.52)

Germination, index, time, rate and vigor represent % germination, germination index, germination time, germination rate and vigor, respectively.

**Table 8:** Protective potential of leaf infusion from the *V. amygdalina*.

Treatments	Germination	Index	Time	Rate	Vigor
**Infected and untreated**					
*Aspergillus flavus*	7.14 (± 10.10)	36.05 (± 3.1)	3.86 (± 0.20)	0.26 (± 0.01)	19.39 (± 27.42)
*Aspergillus niger*	7.14 (± 10.10)	39.77 (± 4.8)	3.79 (± 0.49)	0.27 (± 0.03)	13.27 (± 18.76)
*Aspergillus fumigatus*	0.00 (± 0.00)	32.68 (± 2.3)	3.77 (± 0.15)	0.27 (± 0.01)	0.00 (± 0.00)
*Penicillium* sp.	7.14 (± 0.00)	18.67 (± 5.77)	4.50 (± 0.00)	0.22 (± 0.00)	6.12 (± 8.66)
**Infected, then treated**					
*Aspergillus flavus*	64.29 (± 30.30)	51.52 (± 4.15)	5.44 (± 0.62)	0.19 (± 0.02)	869.4 (± 1021.7)
*Aspergillus niger*	71.43 (± 0.00)	74.73 (± 6.06)	4.90 (± 0.08)	0.20 (± 0.00)	994.9 (± 7.22)
*Aspergillus fumigatus*	64.29 (± 10.10)	62.54 (± 3.23)	5.09 (± 0.19)	0.20 (± 0.01)	638.8 (± 164.5)
*Penicillium* sp.	57.14 (± 20.20)	63.15 (± 9.61)	4.79 (± 0.06)	0.21 (± 0.00)	545.9 (± 382.4)
**Treated, then infected**					
*Aspergillus niger*	0.00 (± 0.00)	33.45 (± 4.55)	3.00 (± 0.71)	0.34 (± 0.08)	0.00 (± 0.00)
*Aspergillus flavus*	0.00 (± 0.00)	0.00 (± 0.00)	0.00 (± 0.00)	0.00 (± 0.00)	0.00 (± 0.00)
*Aspergillus fumigatus*	0.00 (± 0.00)	15.30 (± 4.81)	2.85 (± 0.49)	0.36 (± 0.00)	0.00 (± 0.00)
*Penicillium* sp.	0.00 (± 0.00)	8.93 (± 4.21)	0.00 (± 0.00)	0.00 (± 0.00)	0.00 (± 0.00)
**Infected, then steeped in water**					
*Aspergillus niger*	14.29 (± 20.20)	41.56 (± 1.50)	4.16 (± 0.80)	0.24 (± 0.05)	38.78 (± 54.84)
*Aspergillus flavus*	21.43 (± 10.10)	46.36 (± 6.01)	4.49 (± 0.58)	0.22 (± 0.03)	57.14 (± 51.95)
*Aspergillus fumigatus*	28.57 (± 0.00)	38.66 (± 11.4)	5.27 (± 1.03)	0.19 (± 0.04)	53.06 (± 28.86)
*Penicillium* sp.	14.29 (± 20.20)	41.50 (± 16.9)	4.00 (± 0.40)	0.25 (± 0.03)	26.53(± 37.52)

Germination, index, time, rate and vigor represent % germination, germination index, germination time, germination rate and vigor, respectively.

### Protective potential of the leaf infusions

The okra seeds were soaked in cell suspensions of *Aspergillus niger, A. flavus, A. fumigatus* and *Penicillium* sp. showed no germination throughout the planting period. All fungal pathogens were recorded to inhibit the growth of the okra seeds and initiate infection. No germination was observed for seeds that were steeped in the suspensions of the fungal pathogens without treating with the infusions. The seeds that were infected and then treated with leaf infusions were observed to show remarkable germinability potentials. Control seeds that were infected and then steeped in water before planting remained infected throughout the period of germination, hence showed minute or no germinability potential throughout the planting period (Tables 6, 7 and 8).

## Discussion

In this study, seeds were first steeped in the extracts before planting. Priming of seeds affects the germination of the seeds, thus facilitates their germination^9^. During steeping, seeds have the access to adequate moisture required for their fastidious growth^17,18^. Effect of priming okra seeds with different plant extracts before sowing has significant influence on the germination potential^19^.

From the findings of this study, germinability of the okra seeds was dependent on concentration of the leaf infusion that was used for steeping. In a study by Kiran et al.^20^ 20% was shown to have the highest concentration for the aqueous extract of seeds of *Psoralea corylifolia* on germination and vigor of maize seeds*.* Also, in a study by El-Dahab et al.^21^ when analyzing the plant extracts used on seed viability of sorghum seeds during storage period, 25% concentration was observed to be optimum and more effective than 50, 75 and 100% respectively. Ghasemi et al.^22^ indicated that high concentration of aqueous extract of *Calotropis procera* significantly decreased the germination percentage.

In a study on the effect of *Moringa oleifera* leaf, bark and root extracts on germination and growth of *Triticum aestivum*, optimum concentration that enhanced germination was observed to be 50%^23^. The present study revealed optimum concentration of the *Moringa oleifera* leaf infusion to be 60%. In a related study, Chukwuka et al.^24^ reported that 100% w/v of *Vernonia amygdalina* and *Tithonia diversifolia* produced lower or equal germination percentage, when compared with a control setup in the treatment of *Zea mays* seeds. In a study on the effect of aqueous leaf extract of *Octimum basilicum* and *Artemista absinthium* on tomato seed^25^, tomato seeds treated with aqueous leaf extract of *O. basilicum* and *A. absinthium* had effect on the germination of the seeds. The study revealed that highest germination was observed in seeds treated with either 40% of the *O. basilicum* or 67% of the *A. absinthium* leaf extracts.

Also, a study on the evaluation of *Tithonia diversifolia* for allelopathic effects on some selected crops under laboratory and screen house conditions showed that 100% concentration of the extract lowered germination of soybean seeds^26^. In addition, Aslam et al.^27^ mentioned that lower concentrations of extracts were similar to control and higher concentration inhibited seed germination. The inhibitory effect of extracts on seed germination and growth has also been reported to be dependent on the extract concentration, as the degree of inhibition increases with increase in the extract concentration.

The present study revealed that steeping duration of the okra seeds in the leaf infusions also has effect on germinability. Similar observation has been reported by Akpor and Oluba^28^. It is reported that steeping time has effect on vigor enhancement in seeds. Saleem et al.^29^ reported that 720 min of soaking seeds in water improved the seed vigor of bitter gourd cultivar. For the leaf infusions, steeping duration of 6 h was observed to be optimum when seeds were steeped in *P. bigblobosa* or *V. amygdalina* and 5 h when steeped in *M. oleifera.* Optimum steeping durations of between 5 and 6 h has been reported by Akpor and Oluba^28^ when investigating germinability potential and protective effect of several leaf decoctions against fungal pathogens of *Abelmoschus esculentus.* In addition, Hala et al.^30^ when investigating the effect of *Moringa oleifera* leaf extract on pepper seed germination for seedling improvement, growth, fruit yields and quality, reported optimum soaking time of between 3 and 6 h.

In this study, seeds that were soaked in the leaf infusions before planting showed better germinability potentials than seeds that were only steeped in water before planting. A similar observation was reported by Adejobi and Torjir^31^. Previous study by Murungu^32^, on the effect of seed priming and water potential on seed germination and emergence of wheat varieties in laboratory assays and in the field, seeds that were soaked for 20 and 24 h recorded lowest germination rate. Akpor and Obeasor^15^, have reported optimum soaking time of *Cajanus cajan* in *Helianthus annus* extracts to be 3–4 h. Zhao et al.^33^ recorded that *Hylocereu sundatus* seeds soaked in water for 48 h higher showed higher germination than seeds soaked for 24 h. Although optimum soaking time is necessary to obtain maximum germination, prolonged soaking of seeds has been reported to have negative effect on seed germination^34^.

In this present study, seeds were steeped in suspended cells of *Aspergillus niger, A. flavus, A. fumigatus* and *Penicillium* sp. All the fungal pathogens used for this study were observed to initiate infections on the okra seeds. Results from this study also suggest a possible similar suppressive effect on bacteria pathogens of okra seeds steeped in the test plant infusions as supported by the work of Radwan et al.^35^ will treating barley and wheat seeds with extracts of *Calotropis pocera*. *Aspergillus niger* and *Penicillium chrysogenum* have been recorded to affect the germination and morphology of maize seeds^36^. Gupta et al.^37^ stated that the presence of *Aspergillus* species decreased the germination of seeds and also led to pre- and post-mortality of the seeds. *Aspergillus* species have also been reported to cause significant loss in the seed quality and nutrient content of grains^38^. *Aspergillus* sp. and *Penicillium* sp. are fungal pathogens that are known to produce diverse toxic substances that can affect plants, animals and humans.

In this study, all leaf infusions of *P. bigblobosa, M. oleifera* and *V. amygdalina* were effective against the pathogenic fungi with high germination percentage and vigor index. In a related study by John et al.^39^ reported that *V. amygdalina* leaf powder was effective against fungi associated with infected tomato seeds. In another study, *M. oleifera, V. amygdalina* and *Annona muricate* have been implicated to be effective against *Collectotrichum destructivum* on cowpea seeds^40^. Low concentrations of extracts of *M. oleifera* and *V. amydalina* have been reported to be effective against fungal pathogens^41^. Acid extracts of *Salvia sclarea, S. officinalis* and *Rosmarinus officinalis* have also been reported to be active against *Alternaria*^42^. The various inhibitory effect observed for the target fungal pathogens in this current study may be attributed to the phenolic and flavonoid contents or other bioactive compounds of the various plant infusions^35^. A possible efflux of phytotoxic volatiles from the okra seeds may also cause similar effect and afford the seeds a healthier condition for effective growth^43^.

In a study carried out on the efficacy of plant extracts, traditional materials and antibacterial chemicals against *Xanthomonas campestris* on tomato seed, it was observed that soaking of tomato seeds in mustard and ginger rhizome extracts and lemon juice inhibited seed borne pathogen. Bonzi et al.^44^ reported that seeds steeped for longer duration in aqueous extract of *Balanites aegyptiaca*, *Cymbopogon citratus*, *Cassia occidentalis* and *Portulaca oleracea* inhibited fungal activities against the seeds.

## Conclusion

The findings from this study observed that the leaf infusions from the test plant species positively suppressed the pathogenic treatments and improve the germinability of the test okra seeds. This could only suggest the extracts have potentials to be explored as primers for seed germinability and protection. Although the study interestingly showed positive findings, there is still the need for further investigation in green house and field studies outside this present in vitro report. In addition, a better understanding of the inhibitory compounds in the infusion is necessary for their effective exploitation in seed treatment and germination strategies. These findings can serve as one of the baseline studies for the use of the leaf infusions of indigenous test plant species of ethnobotanical values as alternatives to chemical treatment of seeds.

## Materials and methods

### Preparation of leaf infusions

The plants used for the study the leaves of *Parkia bigblobosa*, *Moringa oleifera* and *Vernonia amygdalina* obtained from the Teaching and Research Farm of Landmark University, Omu-Aran, Kwara State, Nigeria. The plant materials used in the study were identified at the University of Ilorin Herbarium, Ilorin, Nigeria. The voucher specimens of the plant materials were deposited in the Herbarium, with assigned voucher numbers as UILH/001/1023/2021 (*Vernonia amygdalina*), UILH/002/1011/2021 (*Moringa oleifera*) and UILH/003/948/2021 (*Parkia bigblobosa*). The relevant international, national and institutional guidelines were complied with when working with the plants.

The collected leaves were rinsed with water, surface-sterilized following standard practice, and then pulverized using a laboratory blender before steeping in known volumes of water for 24 h. After steeping, the solution was filtered with a muslin cloth. The filtrate (infusion) was discarded while the supernatant was stored in clean air tight transparent plastic bottles at 4 °C. The phytochemical screening of the infusions was carried out using standard procedures^16^.

### Seeds preparation and experimental setup

The okra (*Abelmoschus esculentus*) seeds used for the study were purchased from a local market in Omu-Aran, Kwara State, Nigeria. Prior to use their use, they were soaked in distilled water and the floating ones which were not viable were removed. The viable seeds were surface-sterilized by soaking in sodium hypochlorite (5%) solution for 3 min before rinsing with distilled water several times to remove any residual sodium hypochlorite. These were later steeped in known concentrations for a fixed time. After steeping, seven seeds were drawn and planted in plastic containers (9 cm diameter and 4 height) containing 3.14 g of absorbent cotton wool (to serve as blotters) and incubated under fluorescent light in the laboratory for 7 day for daily observation. The seeds were watered daily to avoid the blotters dehydration.

Germination readings were taken daily, while plant height reading was observed at the end of the 7-d planting period, after which germination time, vigor index, germination rate, germination index values were calculated as follows:

$$ Vigor \, index \, = \, plant \, height \times \, \% germination $$

$$ Germination \, time = \frac{{\left( {G1 \times d1} \right) + \left( {G2 \times d1} \right) + \left( {G3 \times d3} \right) + \cdots \left( {G7 \times d7} \right)}}{G1 + G2 + G3 \ldots G7} $$

$$ Germination \, rate = \frac{G1 + G2 + G3 + \cdots G7}{{\left( {G1 \times d1} \right) + \left( {G2 \times d2} \right) + \left( {G3 \times d3} \right) \ldots \left( {G7 \times d7} \right)}} $$

where G and d represent % germination and the day of planting respectively.

All experimental setups were carried out in duplicate. A total of seven seeds were used per per treatment.

### Determination of optimum concentration

For the determination of the optimum concentration, five different concentrations of the respective leaf infusions were used. The concentrations were 0:5, 1:5, 2:5, 3:5, 4:5 and 5:5 volumes of the respective leaf infusion ratio to volume of water. The surface-sterilized seeds were steeped in the respective concentrations for 1 h before planting and daily observation, as described earlier.

### Determination of optimum soaking time

For optimum steeping duration, the surface-sterilized seeds were steeped in a known concentration of the respective leaf infusions. Every 1 h for a duration of 6 h, 7 seeds were withdrawn from each of the setup and planted in transparent plates as described earlier. Daily germination of seeds was recorded. At the expiration day, plant height, germination rate, vigor index, % germination of each of the slots was estimated as described earlier.

### Assessment of protective potential of the infusions

The protective potential of the infusions against selected fungal pathogens was observed, using the optimum concentration of the respective infusions and the steeping duration that gave better seeds’ germinability results. The fungal pathogens used for the experiment were *Aspergillus niger*, *A. flavus*, *A. fumigatus* and *Penicillium* sp. The isolates were already subcultured in sabouraud dextrose broth at 25 °C for 72 h.

Five experimental setups were used for this study:

- Seeds were soaked in respective plants infusions only,
- Seeds soaked in broth cultures of the respective fungal pathogens,
- Seeds that were soaked in broth cultures of the fungal pathogens before soaking in infusion,
- Seeds soaked in broth cultures of the fungal pathogens before soaking in distilled water, and
- Seeds soaked in leaf infusions before soaking in broth cultures of fungal pathogens.

Except for seeds that were untreated, which were soaked for 1 h duration, the treatment setups (leaf infusion or water) were first soaked for 1 h in the fungal suspension before soaking for 1 h in the treatment fluid. Seeds that were first treated before infecting with the pathogens were soaked in the treatment solution (leaf infusion or water) before transferring to the fungal suspension for another 1 h. Seeds were planted and monitored as described earlier.

### Statistical analysis

Descriptive statistics and analysis of variance (ANOVA) were carried out using SPSS (version 23) statistical package. Treatment means were compared with Duncan’s multiple range test at p = 0.05 level of significance.
